# Current insights in ultra-rare adenylosuccinate synthetase 1 myopathy – meeting report on the First Clinical and Scientific Conference. 3 June 2024, National Centre for Advancing Translational Science, Rockville, Maryland, the United States of America

**DOI:** 10.1186/s13023-024-03429-x

**Published:** 2024-11-26

**Authors:** Emma Rybalka, Hyung Jun Park, Atchayaram Nalini, Dipti Baskar, Kiran Polavarapu, Hacer Durmus, Yang Xia, Linlin Wan, Perry B. Shieh, Behzad Moghadaszadeh, Alan H. Beggs, David L. Mack, Alec S. T. Smith, Wendy Hanna-Rose, Hyder A. Jinnah, Cara A. Timpani, Min Shen, Jaymin Upadhyay, Jeffrey J. Brault, Matthew D. Hall, Naveen Baweja, Priyanka Kakkar

**Affiliations:** 1 Institute for Health and Sport, Victoria University, Melbourne, VIC Australia; 2 Inherited and Acquired Myopathies Program, Australian Institute for Musculoskeletal Science, St Albans, VIC Australia; 3 Department of Neurology, Gangnam Severance Hospital, Yonshei University College of Medicine, Seoul, Republic of Korea; 4 Department of Neurology, National Institute of Mental Health And NeuroSciences (NIMHANS), Bengaluru, India; 5 Children’s Hospital of Eastern Ontario Research Institute, Ottawa, K1H 5B2 Canada; 6 Department of Neurology, Istanbul Faculty of Medicine, Istanbul University, Istanbul, Turkey; 7 Xiangya Hospital, National Medical Metabolomics International Collaborative Research Center, Central South University, Changsha, China; 8 Department of Radiology, Xiangya Hospital of Central South University, Changsha, China; 9 Departments of Neurology and Pediatrics, University of California Los Angeles, Los Angeles, USA; 10 Division of Genetics and Genomics, The Manton Centre for Orphan Disease Research, Boston Children’s Hospital, Harvard Medical School, Boston, MA USA; 11 Institute for Stem Cell and Regenerative Medicine, University of Washington, Seattle, WA USA; 12 Department of Biochemistry and Molecular Biology, The Pennsylvania State University, University Park, PA USA; 13 Departments of Neurology, Human Genetics and Pediatrics, Emory University, Atlanta, USA; 14 Division of Preclinical Innovation, National Centre for Advancing Translational Science, National Institutes of Health, Rockville, MD USA; 15 Department of Anaesthesia, Critical Care and Pain Management, Boston Children’s Hospital, Harvard Medical School, Boston, MA USA; 16 Department of Psychiatry, McLean Hospital, Harvard Medical School, Belmont, MA USA; 17 Center for Musculoskeletal Health, Department of Anatomy, Cell Biology & Physiology, Indiana School of Medicine, Indianapolis, IN USA; 18 Cure ADSSL1, Glendale, CA USA

**Keywords:** Adenylosuccinate synthetase 1 myopathy, ADSS1 myopathy, Inborn error of metabolism, Purine disorder, Ultra-rare neuromuscular disease, Skeletal muscle, Cardiac muscle, Clinical presentation, Pre-clinical models, Therapeutics, Biomarkers, Consortium, Guidelines

## Abstract

The inaugural Clinical and Scientific Conference on Adenylosuccinate Synthetase 1 (ADSS1) myopathy was held on June 3, 2024, at the National Institutes of Health (NIH) National Center for Advancing Translational Sciences (NCATS) in Rockville, Maryland, USA.

ADSS1 myopathy is an ultra-rare, inherited neuromuscular disease.

Features of geographical patient clusters in South Korea, Japan, India and the United States of America were characterised and discussed.

Pre-clinical animal and cell-based models were discussed, providing unique insight into disease pathogenesis.

The biochemical pathogenesis was discussed, and potential therapeutic targets identified.

Potential clinical and pre-clinical biomarkers were discussed.

An ADSS1 myopathy consortium was established and a roadmap for therapeutic development created.

## Introduction

Adenylosuccinate synthetase 1 (ADSS1) myopathy is an ultra-rare, slowly progressive neuromuscular disease (NMD) leading to progressive deterioration and loss of function of skeletal and cardiac muscles. Fifty-two representatives of academia, clinics, patient organisations and industry from eight countries (Australia, China, Germany, India, South Korea, India, Turkey, United States of America and Wales) attended the meeting organised by Cure ADSSL1, a foundation dedicated to advocating for patients with ADSS1 myopathy and discovering treatments. The meeting was hosted at the National Institutes of Health (NIH), National Center for Advancing Translational Science (NCATS). Eighteen delegates attended in-person and thirty-four attended on-line.

The aim of the meeting was to unite Cure ADSSL1’s research and clinical collaborators into a focused discussion on disease presentation, pathogenesis and biomarkers, and the pathway to therapeutic development.

The meeting commenced with welcome remarks by Naveen Baweja and Priyanka Kakkar, Founder, and Founder and President (respectively), of patient organisation Cure ADSSL1, and Dr. Matthew Hall, Scientific Director of NCATS. The daylong meeting was comprised of four topical sessions: (1) Clinical characteristics of ADSS1 myopathy patients, (2) Insights from animal and cell models, (3) Small molecule targets and therapeutics, and (4) Biomarker discovery. Presentations within the clinical session were delivered by international clinicians attending on-line, so a clinic-focused discussion session was hosted immediately after Session One by Dr Perry Shieh. Post-presentation discussions were initiated, and a generalised meeting discussion was hosted at the conclusion of the meeting by Cure ADSSL1’s, Naveen Baweja.

### Background

**Fig. 1:**
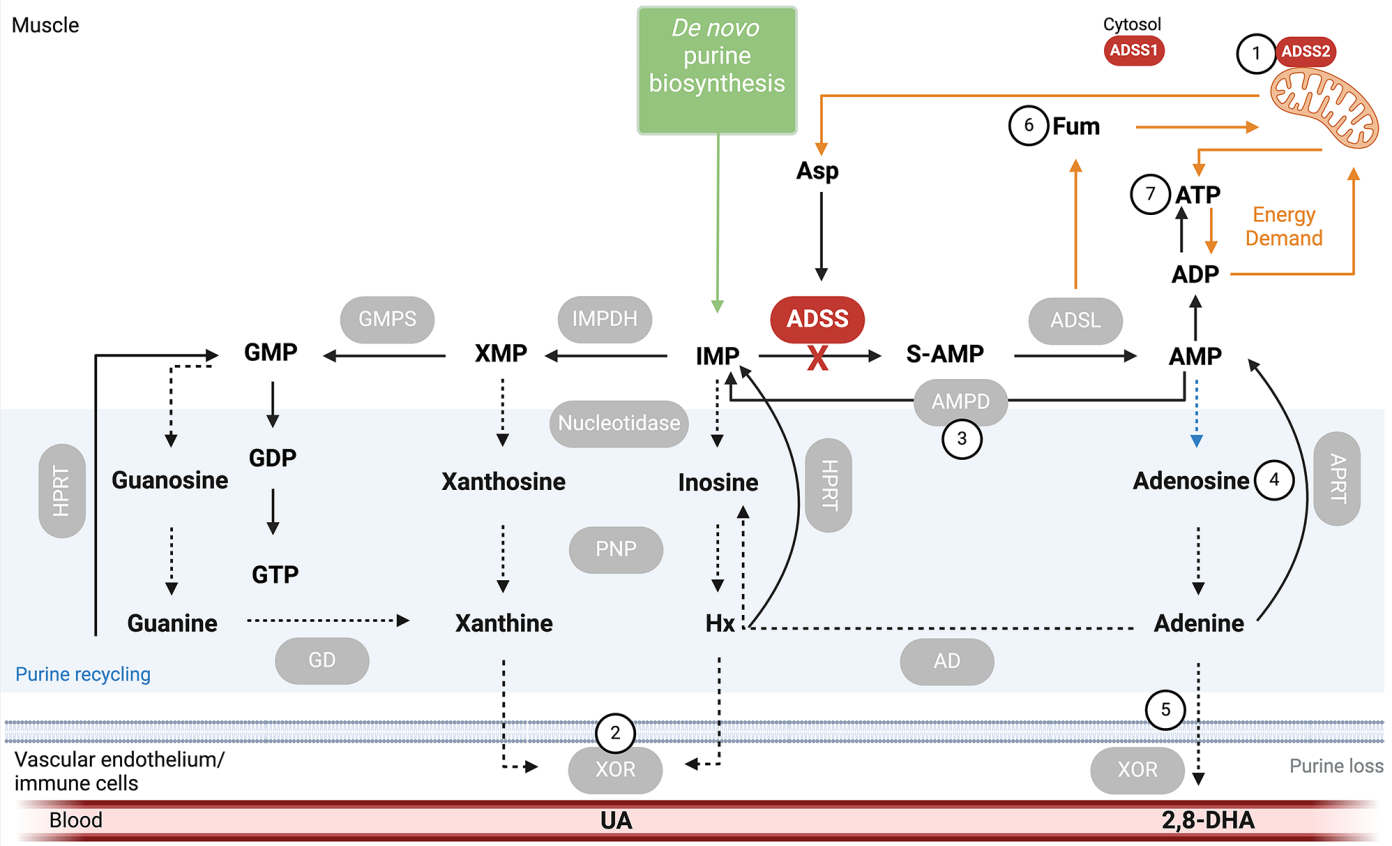
Location and metabolic activity of Adenylosuccinate Synthetase 1 (ADSS1) within purine metabolism. Loss of ADSS1 function compromises the conversion of inosine monophosphate (IMP), generated by *de novo* purine biosynthesis and adenosine monophosphate deaminase (AMPD), to succinyl-AMP/adenylosuccinate (S-AMP) and adenosine monophosphate (AMP). Energy production capacity may be reduced. IMP may be diverted into other reactions and/or degraded and excreted from muscle leading to purine depletion. Potential targets for restoration of metabolic systems are indicated in numbered circles: (1) Overexpression of mitochondrial ADSS2; (2) Inhibition of xanthine oxidoreductase (XOR); (3) Inhibition of AMP deaminase (AMPD); (4) Targeting recycling pathways to increase adenosine concentration; (5) Supplemental adenine combined with XOR inhibition; (6) Supplemental fumarate esters; (7) Supplemental ATP. Schematic was created with Biorender.com

First described in 2016 by Park and colleagues in a small cohort of Korean patients clinically presenting with distal muscle weakness [1], ADSS1 myopathy is an inborn error of metabolism caused by autosomal recessive mutations of the *ADSS1* gene, which encodes a striated muscle-specific isoform of adenylosuccinate synthetase (ADSS1). There are 124 genetically diagnosed cases spread across 5 countries, although it is estimated (based on 2024 Korean incidence per capita) that there could be as many ~ 4419 ADSS1 myopathy patients worldwide, mostly of Asian descent. Disease clusters have been identified in Korea [1, 2] and Japan [3]. ADSS1 functions within the purine metabolic pathway involved in maintaining purine nucleotide concentrations in muscle (summarised in Fig. 1).

The classical phenotype is a slowly progressive myopathy with diffuse muscle weakness that typically starts in childhood and is followed by progressive muscle weakness in adolescence when the disease is usually diagnosed [4]. Although originally described as a distal myopathy, there is extensive proximal (e.g., quadriceps) muscle involvement and facial muscle weakness in most patients. Cardiomyopathy and respiratory deficiency is also common in advanced disease with death frequently caused by respiratory failure. Muscle histopathology may include lipidosis, internalised nuclei, fibre splitting, sarcomere disorganisation and focal fibrosis, sometimes with nemaline rods (particularly in Japanese patients) [3]. However, disease trajectory is highly variable between patients. There is an urgent need to understand the pathogenesis of the disease to inform clinical management and therapeutic development. This was a focus of several presentations within the meeting.

Cure ADSSL1 is the only organisation worldwide exclusively focused on supporting and advancing treatments for ADSS1 myopathy patients. Priyanka Kakkar and Naveen Baweja founded the organisation in 2021 after their two children were diagnosed with the disorder. The work of Dr. Park reporting the first cases of ADSS1 myopathy provided the community with an important knowledge resource to learn from. The Cure ADSSL1 founders quickly realised that without intensive research effort across the discovery, pre-clinical and clinical landscapes, an effective treatment was unlikely to become available to their children within their lifetimes, if ever. They formed Cure ADSSL1 and began connecting with patients, academics and industry partners to characterise the disease and develop treatments for ADSS1 patients worldwide (https://www.cure-adssl1.org/) [5].

The meeting was transcribed to provide the rare NMD community with a current account of published and preliminary data on ADSS1 myopathy.

## Clinical perspectives

### Session 1: Clinical characteristics of ADSS1 myopathy patients

Dr. Hyung Jun Park, M.D., Ph.D. presented clinical characteristics of the Korean ADSS1 myopathy patient cohort. Dr. Park’s team was the first to discover a family (3 patients) presenting with juvenile onset distal myopathy presenting as weakness and wasting with mild facial muscle involvement [1]. Muscle biopsy revealed rare fibres with rimmed vacuoles [4]. Whole exome sequencing on affected and unaffected family members revealed biallelic predicted damaging variants in *ADSS1*. However, *ADSS1* was not considered causal of myopathy until the same variants were also detected in a non-related patient presenting with similar symptoms [1]. Consequently, Dr. Park’s team hypothesised loss of ADSS1 function and purine homeostasis to be the cause of myopathy. Knockdown of ADSS1 in murine myoblasts using RNA silencing reduced myoblast but not fibroblast viability. Injection of ADSS1 morpholino into zebrafish to knockdown endogenous ADSS1 caused misshapen embryos and loosely packed

fibres with occasional prominent gaps. Co-injection of human normal ADSS1 mRNA rescued the zebrafish phenotype [1]. Transcriptome analysis of skeletal muscle biopsies from ADSS1 myopathy patients revealed significant downregulation of all three purine nucleotide cycle (PNC) genes: ADSS1, adenylosuccinate lyase (ADSL) and adenosine monophosphate deaminase 1 (AMPD1) [6]. PNC metabolism may be especially crucial during strenuous exercise or starvation when the ATP reservoir runs low and disruption probably leads to muscle dysfunction during exercise or other energy-stressing scenarios [7, 8].

The current Korean patient cohort comprises 28 patients with an average age of presentation of 8 years (6.8–14.5 years) and current mean cohort age of 34.5 years (24.8–44 years). Of the cohort, 61% are male and 39% are female. 93% of patients carried one of two pathogenic variants: NM_152328.5:c.781G > A, p.Asp261Asn and NM_152328.5:c.919del, p.Ile307SerfsTer25 variants account for 52% and 41%, respectively. Of the 28 patients, 19 have predominantly distal weakness, 5 have predominantly proximal weakness, 4 have equal involvement of proximal and distal muscles and 1 has no weakness. There is facial muscle involvement in 22 patients, 17 report rapid fatigability on exertion, 17 manifest dysphagia with masticatory dysfunction and 12 manifest dysarthria. Normal motor milestones were reached during infancy. Most self-reported as slow runners during childhood, with frequent falling and steppage gait due to bilateral foot drop during adolescence. Fourteen patients are ambulatory with assistance at an average age of 17 years (10.5–24 years). Eleven are ambulatory with a walking aid at an average age of 28 years (21.5–33 years) and 3 patients are non-ambulatory at an average age of 47 (44-50.5 years). The last ambulatory age of the non-ambulatory patients was 42-, 55- and 38-years following disease durations of 35, 37 and 32 years, respectively. All except the one patient without muscle weakness has bi-facial weakness, reports to sleep with eyes open and has an expressionless, youthful face from puberty. Most patients over 30 years complained of nasal voice, and one third of patients have difficulty chewing and dysphagia. These symptoms worsen slowly with age. 14% of Korean ADSS1 myopathy patients have ventricular hypertrophy compared to 25% of patients in the Japanese cohort [3]. 21% of Korean patients have reduced vital capacity and exertional dyspnoea, although none required mechanical ventilation. Comparatively, 41% of Japanese patients have marked reduction of vital capacity irrespective of age, with 11% necessitating mechanical ventilation [3]. Histology has been assessed in 14 of the 28-patient cohort (50%). Variation of fibre size, and type I fibre predominance with rimmed vacuoles and nemaline bodies is evident. Nemaline bodies are especially found around type I fibres. Fatty replacement of especially tongue and masseter muscles is frequently observed in patients using magnetic resonance imaging (MRI). Arm muscles are frequently spared at early stages of disease but progressively degenerate in advanced disease.

Two distinct Korean ADSS1 myopathy cases were presented with notably marked differences in disease trajectory observed within the same family. A 32-year-old female and 30-year-old male sibling reported feeling weaker than peers around 8 years of age and experienced steppage gait at 14 years. There were no significant differences in strength between the siblings at age 20. The younger male began strength training and noticed a decline in strength. Thereafter, the male patient progressed more rapidly than the female sibling and lost ambulation. The older sister remains ambulatory. There may be a relationship between exercise and disease severity although this is difficult to determine based on this single family. Further, a 20-year-old male patient reported post-exercise muscle pain and rhabdomyolysis in the past 3 years. Neurological examination revealed normal muscle strength and ability to jump. Serum creatine kinase (CK) levels were typically 320 U/L but increased to 8520 U/L after exercise. Exome sequencing identified a c.919del homozygous variant. The patient has been monitored for > 1 year and muscle strength remains normal. A non-ischemic forearm test on the same patient revealed normal elevation of ammonia and lactate, although these data might be explained by muscle weakness confounding the tests results. Further clinical studies are required to identify the relationship between genotype and phenotype and the underlying pathomechanisms.

Professor Atchayaram Nalini, M.D., Ph.D. and Dr Dipti Baskar, M.D. presented on the clinical characteristics of the current Indian patient cohort. The pooled prevalence of ADSS1 myopathy in India based on data from genetic laboratories is 28 patients. *ADSS1* variants were classified as per ACMG guidelines and annotated based on muscle-specific reference sequence transcript NM_152328.5. Based on whole exome sequencing, the most common variant is c.781G > A (p.Asp261Asn). Other variants including c.794G > A, p.(Gly265Gly) have been identified. The characteristic clinicopathologies of 6 patients (4 females, 2 males) have recently been published in a retrospective descriptive study conducted between 2016 and 2022 ^9^. Baseline demographic details and laboratory findings, including muscle MRI and histopathology, were collected from the medical records and analysed. All patients had onset of symptoms in the first decade, with a mean age at presentation of 17.7 ± 8.4 years (range 3–27 years). All patients had slow walking and difficulty running. Four patients had significant limb fatigability, two patients had dysphagia with chewing difficulty, three patients had exertion-induced palpitations, three patients had eyelid ptosis and one had divergent squint. Serum CK levels were mildly elevated above normal (i.e., 20–200 U/L) in one male (1564 U/L) and one female (932 U/L) patient only. MRI revealed fatty infiltration of the gluteus maximus, gastrocnemius and soleus, and gluteus maximus and semimembranosus muscles in two male patients, respectively. Muscle biopsy was performed in two patients: intracellular lipid vacuoles were seen in the female while rimmed vacuoles and nemaline rods were seen in the male. All, except one female who had a c.794G > A, p.Gly265Glu variant, which has been previously reported within the Japanese [3] and Korean [1] cohorts, had the pathogenic c.781G > A, p.Asp261Asn variant. Clinical evaluation showed elongated myopathic face of a 15-year-old male patient, absent nasolabial folds in a 33-year-old male patient, atrophic scar of the skin in a 27-year-old patient and distal laxity of joints in a 21-year-old female patient. Repetitive nerve stimulation studies were performed in five patients of whom two siblings in the first family showed significant decremental response (15–20% in trapezius and facial muscles). Mild QTc prolongation and biventricular dysfunction were observed in one female patient.

*Clinical progression and treatment.* Patient 1 is a 21-year-old female with onset from 4 years age (17-year illness duration who walks with minimal support although reports dyspnea on walking short distances and fatigability). The patient is currently treated with Coenzyme Q10, Vitamin E, Vitamin D, Levocarnitine and ATP. Patient 2 is a 17-year-old male with disease onset at 1 year (16-year illness duration) who has used an ankle foot orthosis since 15-years-age and reports fatigability. There is hyperuricemia and hypercalciuria. The patient is currently treated with urocit-K, allopurinol and pyridostigmine (. Patients 1 and 2 are siblings. Patient 3 is a 33-year-old female with onset of symptoms at 13 years (20-year illness duration) and symptoms including progressive fatigability, difficulty rising from the floor and walking a few steps without support for ~ 3 years, and persistent dysphagia. Current treatments include multivitamins and initial treatment with pyridostigmine (60 mg/day), which although significantly improved fatigue and strength, caused severe diarrhoea resulting in treatment cessation. Patient 4 is a 40-year-old male with onset of symptoms at 17 years (23-year illness duration). He uses a wheelchair and BIPAP machine nocturnally and for 2–3 h/day to assist ventilation. He has been unable to work for ~ 7 months and experiences fatigue. He is currently being treated with telmisartan (40 mg/day) and carvedilol (25 mg/day) for cardiomyopathy. Patient 5 is a 5-year-old female with disease onset at 1 year age (4-year illness duration). The patient has been treated with ATP disodium for 2 years. Patient 6 is a 22-year-old female with disease onset at 4 years (18-year illness duration). She has used a wheelchair and nocturnal BIPAP respiratory support for 13 years. She has progressive dysphagia and chewing fatigue. She is currently treated with CoQ10, Vitamin B2, Vitamin D, and occasionally pyridostigmine and creatine.

Unpublished clinical and histopathology (light microscopy) data from other patients of Indian origin have been collected with the support of Cure ADSSL1. A 39-year-old male patient (homozygous c.781G > A) with disease onset at 5 years has proximal upper and lower leg weakness and cardiomyopathy. Muscle MRI shows fatty infiltration of the hamstrings, peroneal and posterior leg compartments. There is oedema in the anterior thigh and distal calves. Muscle biopsy shows vacuolated fibres with glycogen aggregates (periodic acid Schiff (PAS) positive) with partial diastase resistance (indicating polyglucosans), and several cytochrome c oxidase (COX)-deficient fibres. A 32-year-old female patient (homozygous c.781G > A) with disease onset at 28 years shows fatigable weakness of the lower limb and pelvic muscles. Muscle biopsy shows fibre size variation with clear vacuoles (PAS+, diastase sensitive), rimmed vacuoles (mGT, p62 and TDP43 + aggregates), subsarcolemmal basophilic pyramidal inclusions (COX/NADH negative) with myocyte necrosis and phagocytosis. A 65-year-old male patient (homozygous c.781G > A) with disease onset at 17 years initially showed fatigable weakness of proximal lower limb muscles with progression to upper limb and ptosis. He became wheelchair bound at 40 years of age and has used non-invasive ventilation and a feeding tube since 45- and 60-years-of-age, respectively due to progressive dysphagia. Muscle biopsy showed small angulated atrophic fibres and hypertrophied lobulated fibres with central nuclei (dystrophic and neurogenic changes). A 14-year-old male patient (homozygous c.781G > A) with disease onset at 8 years has lower limb weakness. A 51-year-old male patient (homozygous c.781G > A) with disease onset at 4 years has lower limb weakness, assisted ambulation since 44-years-age and nocturnal CPAP assisted ventilation since 46-years-age. A 26-year-old male patient with disease onset at 16 years has lower limb weakness and cannot walk further than 750 m unassisted.

Salient features of the Indian ADSS1 myopathy cohort are a common c.781G > A, p.Asp261Asn variant with onset of symptoms in the first or second decade of life. The disease is steadily progressive with requirement of assisted ambulation by the 4th -5th decade. Most patients develop features of neuromuscular junction impairment, fatigable chewing with dysphagia, nocturnal respiratory failure requiring CPAP/BIPAP and cardiac involvement (cardiomyopathy/rhythm abnormalities). There is prominent muscle fatigability and ptosis that responds to pyridostigmine. Muscle MRI shows fatty infiltrations of posterior compartments of thigh and legs, and muscle biopsy shows intracellular vacuoles (often rimmed) containing lipids or glycogens.

Dr Hacer Durmus M.D., Ph.D. presented on the one diagnosed Turkish patient to date. The case study was published in 2020 ^10^. The patient presented at 18-years-age with difficulty in climbing stairs, proximal muscle weakness of the triceps, iliopsoas, gluteus maximus and quadriceps femoris muscles, and mild orbicularis oculi and lower facial weakness. They reported being slower than peers and easily fatigable. The patient presented with facial weakness, high arched palate, generalised muscle atrophy, severe contractures, rigid spine, diffuse muscle weakness with proximal dominance but no tongue weakness. Serum CK levels were 4–8 times higher than reference values and lactate was mildly elevated. Electromyography (EMG) revealed a chronic myopathic pattern, echocardiography and 24-hour Holter monitoring were normal and there were no respiratory complaints. Muscle biopsy showed myofibers with internalised nuclei, rod-like inclusions, and rare rimmed and unrimmed vacuoles. Muscle MRI showed proximal dominant fatty infiltrations especially in the quadriceps and vastus intermedius muscles. The patient was born to a consanguineous family with no other affected family members. Genetic findings revealed a novel homozygous variant c.898G > A, p.Ala300Thr located in the highly conserved *ADSS1* domain that binds inosine monophosphate (IMP). The patient was still ambulatory at 35-years-age, but respiratory deficiency emerged from 30 years. The patient died due to respiratory problems at 38 years age.

### Clinical discussion and action points

Dr Perry Shieh M.D. led the clinical discussion panel. Discussions centred around underdiagnosis of the disease due to *ADSS1* not being included on the standard neuromuscular screening panels. Even when whole exome sequencing is used, pathogenic *ADSS1* mutations may be interpreted as variants of uncertain significance and underreported. Also, in many countries, there are limited commercial genetic screening options that provide diagnostic services for ultrarare neuromuscular diseases.

The variation in symptoms across and within geographical patient clusters (summarised in Tables 1 and 2) was another discussion topic. For example, contractures were experienced by the Turkish patient but have not been reported in other patient clusters. There is an interesting variation in the involvement of distal and proximal muscles across patients, yet both facial muscle weakness and reduction of respiratory function seem to be compromised across all the patients. Muscle histopathology of some patients reveals multiple features reminiscent of mitochondrial disease while others have minor non-specific histopathologic symptoms. There is also significant variability in the severity of symptoms that can lead to differential diagnosis (Table 3). Some patients progress rapidly to loss of ambulation and respiratory insufficiency, while others have milder symptoms. Biological sex may also be a factor. A deeper understanding of the pathophysiological drivers of progressive disease is needed to direct clinical management and therapeutic development.

**Table 1:** Summary of published data on global ADSS1 myopathy patient cohorts

Geographical Location	Cohort *n*	Associated References
Korea	28	[1, 2, 4, 11]
Japan	63	[3, 12]
India	28	[9]
Turkey	1	[10]
USA	4	[13, 14]

**Table 2:** Summary of cohort characteristics as presented during the conference

	Korea									Turkey/ India		USA			India								
Publication	Park et al. 2016^1^				Park et al. 2017a^4^	Park et al. 2017b^2^				Mrocek et al. 2020^10^		Farid et al. 2023^13^			Baskar et al. 2024^9^				Baskar and Atchayaram, unpublished				
ID	1	2	3	4	5	6	7	8	9	1	2	1	2	3	1	2	3	4	5	6	7	8	9
Sex	M	M	M	F	F	M	M	F	M	M	F	M	F	M	F	M	F	M	F	F	M	F	M
Variants	^a, b^	^a, b^	^a, b^	^a, b^	^a, b^	^a, b^	^a, b^	^a, b^	^a, c^	^d, d^	^a, a^	^a, a^	^a, a^	^a, a^	^a, a^	^a, a^	^e, e^	^a, a^	^a, a^	^a, a^	^a, a^	^a, a^	^a, a^
Age at onset	15	13	13	13	8	5	7	7	6	18	5	1	3	17	7	10	13	17	1	13	5	28	17
Disease duration	16	17	15	10	7	24	25	30	28	16	13	13	16	46	12	5	11	10	2	5	34	4	48
**Clinical presentation**																							
Diffuse muscle weakness	+	+	+		+	+	+	+	+														
Ankle dorsiflexor weakness	+	+	+			+			+														
Distal muscle weakness				+			+	+		+	+	+											
Proximal muscle weakness/atrophy (quadriceps)							+	+		+			+		+	+	+	+	+	+	+	+	+
Generalised muscle atrophy										+													
Tripping																	+			+			
Facial muscle involvement	+	+	+	+	+	+	+	+	+	+	+			+			+	+		+	+		+
Serum creatine kinase IU/L, mean SD range	365	271	250	493	281	420	431	108	493	960–1850	150–1410				150	123	129	1564	932	221			
**Muscle histopathology**																							
Rimmed vacuoles	+	+	+															+			+	+	
Chronic myopathy/necrosis				+		+				+												+	
Internalised nuclei										+	+												
Nemaline rods							+											+					
Fibre size variation											+											+	+
Scattered hyaline fibres											+												
Intracellular lipid vacuoles																	+			+			
Polyglucosan aggregates																					+		
Ragged red fibres																				+			
Cytochrome c oxidase deficiency																					+		
Congenital fibre type disproportion																				+			

Key: ^a^c.910G > A/ c.781G > A, ^b^c.1048delA/c.919delA, ^c^c.1220T > C, ^d^c.898G > A, ^e^c.794G > A; + denotes symptoms and histopathologies

**Table 3:** Differential diagnosis of ADSS1 Myopathy from other commonly considered diseases

	Slowly progressive muscle weakness	Distal muscle weakness	Proximal muscle weakness	Facial muscle weakness	Elevated creatine kinase	Reduced nerve conduction	Histopathology- Nemaline rods	References
ADSS1 Myopathy	+	+	+	+	Variable, isolated patients	+ (unpublished observations from Dr. Perry Sheih)	+ (common in Japanese patients)	^3, 4, 9, 15^
Nemaline Myopathy	+		+	+			+	^16, 17^
Metabolic Myopathies	+	+	+	+	+			^18, 19^
Muscular Dystrophies	+	+	+		+			^20^
Myasthenia Gravis	+		+	+		+		^21^

+ denotes symptoms and histopathology

In a roundtable discussion about biomarkers for future clinical trials, Alan Beggs questioned whether correlations have been performed between MRI muscle/fat fraction, serum creatine phosphokinase (CPK) and ambulatory capacity. Atcharyaram Nalini indicated there is low correlation between CPK and weakness and ambulatory capacity. Alan Beggs highlighted that when muscle is replaced with fat, CPK levels will become lower due to lower muscle mass rather than a slowed rate of progression of the myopathy. Hacer Durmus thinks that CPK is a poor biomarker of all NMD’s, and that MRI is probably not an early biomarker of ADSS1 myopathy. Hyder Jinnah thought that under controlled settings, such as before and after a standardised exercise stressor, CPK could be a valuable biomarker test for individual cases and should not be dismissed. Since serum creatinine is correlated with muscle mass, normalisation of CPK to serum creatinine is another approach. Serum/urinary creatinine levels are also purported to be useful biomarkers for muscle and may be clinically useful in the context of ADSS1 myopathy.

Perry Shieh suggested two potential mechanisms that could contribute to fatigue in ADSS1 myopathy patients. While increased Oil Red O-positive histopathology is consistent with a metabolic fuel-deficient myopathy, the positive repetitive nerve stimulation raises the possibility of a defect in neuromuscular transmission. Hacer Durmus thought the metabolic myopathy is probably most influential and that respiratory problems need to be carefully monitored since they appear to be the leading cause of mortality. The cardiac phenotype needs more intensive study also. Perry Shieh indicated that the neuromuscular transmission defect was only seen in tibialis anterior (TA) muscle. He also wondered whether cardiac MRI studies might be more revealing than the more commonly used echocardiography.

Exercise may worsen the clinical phenotype and Hacer Durmus suggested that this is an important consideration. Perry Shieh advises most of his NMD patients (particularly dystrophies) to avoid eccentric and anaerobic (glycolytic) exercise and, if anything, focus more on concentric and aerobic-type exercise. In ADSS1 myopathy, there appears to be a disruption of both glycolytic and oxidative metabolism, so any form of higher intensity exercise may be detrimental. Diet is another important consideration. Perry Shieh questioned whether different diets and activity could contribute to the variation of phenotype, and to especially, anecdotal sex differences in disease presentation. Hyder Jinnah suggested that broad phenotypic variation might teach us something, but it also might just reflect human biological variation, which is very common across many genetic diseases. Jeff Brault wondered how many patients co-express genetic variants in *AMPD* which are very common.

It was agreed that biobanking tissues and sharing across the different international clinics should be pursued.

## Pre-clinical perspectives

### Session 2: Insights from animal and cell models

Professor Yang Xia, M.D., Ph.D. and Dr Linlin Wan, M.D., presented on the metabolomic profiles of ADSS1 deficient mice and patients providing unique insight into the ADSS1 myopathy disease signature in blood and muscle. Professor Xia and Dr Wan acknowledged contributions by the National International Collaborative Research Center for Medical Metabolomics, Professor Rodney Kellems from the University of Texas Houston, USA and Professor Angelo D’Alessandro from the University of Colorado School of Medicine, USA.

Dr Behzad Moghadaszadeh and Professor Alan Beggs presented on two novel mouse models of ADSS1 myopathy. The Beggs Lab is phenotyping (1) an *Adss1* knockout (KO) developed by Cyagen and courtesy of Ling Li (Beckman Research Institute City of Hope), generated by homozygous excision of a floxed exon 2; and (2) an *Adss1* knock-in (KI) developed by Taconic by inducing the most common founder p.Asp261Asn mutation seen in East-Asian and Indian patient cohorts, resulting from a missense change in exon 8.

*Adss1* KO mice were bred to homozygosity, are viable and a late onset phenotype is emerging. Preliminary results from molecular and biochemical analysis of one homozygote were presented. Western blotting confirmed loss of ADSS1 protein in skeletal and cardiac muscles. By quantitative rtPCR both *Adss1* and *Adss2* gene expression appears reduced in skeletal muscles of *Adss1* KO versus normal controls. To test whether expression of the non-muscle ADSS2 isoform may compensate or modulate the effects of ADSS1 deficiency the Beggs lab has generated specific ADSS1 and ADSS2 antibodies (available on request).

The intention of developing a KI model was to induce a milder phenotype relative to the predicted severe phenotype of the KO model. However, paradoxically, homozygous *Adss1*^*p*.Asp261Asn/Asp261Asn^ mice derived from a single Taconic founder exhibit an early neonatal lethal phenotype. Compound heterozygous mice, generated by crossbreeding *Adss1* KO with *Adss1*^p.Asn261Asp^ KIs are systematically smaller and weaker than littermates and have reduced grip strength and fatigue quicker in forced treadmill tests. However, it is unclear whether the lethality/increased severity associated with KI allele is an off-target effect of the CRISPR/Cas9-mediated gene editing, or something else. One possibility is a dominant negative interaction involving heterodimerisation of normal ADSS2 with mutant ADSS1. Genomic sequencing and further breeding studies to evaluate these hypotheses are underway. Clearly there is an urgent need to specify the underlying cause of the KI mutation to ensure the model is human phenotype relevant.

In open discussions, Dr Moghadaszadeh and Professor Beggs acknowledged the important contributions of Dr. Jeff Widrick, and Emily Hickey to their animal experiments and colony management, respectively. The possibility of heterodimerisation between ADSS1 and ADSS2 was discussed. Matt Hall indicated that many times researchers approach him having CRISPR edited a mutation into a gene to induce a disease phenotype, and to request an inhibitor of the enzyme. But when the enzyme is inhibited, the disease phenotype cannot be recapitulated. There is a big difference between totally removing ADSS1 protein and installing a point mutation in a protein that is present. It is not surprising that there is such a phenotype difference between the experimental model approaches. Importantly, we do not know all the things a protein might be doing aside from its core enzymatic activity. Monkol Lek suggested genomic sequencing to determine potential off target effects of the KI mutation, which is presently being pursued.

Associate Professor David L. Mack, Ph.D. and Assistant Professor Alec Smith, Ph.D. presented on patient-derived and CRISPR-edited 3-dimensional engineered cardiac and skeletal muscle tissues for modelling disease and drug discovery. Workflow for developing inducible pluripotent stem cells (iPSC)-based ‘disease-in-a-dish’ prototypes was discussed [22]. Patients come into the clinic and urine is collected, from which urine-derived stem cells (USCs) are isolated and propagated in cell culture and banked to capture the genotype. USCs are then reprogrammed into iPSCs, which are maintained in culture and directed to differentiate into the cell type most likely to manifest the disease phenotype, e.g., cardiomyocytes, skeletal muscle myotubes. Then, the phenotype in the dish is comprehensively characterized to determine whether it manifests the same symptoms as patients and to identify the earliest molecular drivers of pathology from a developmental perspective. Correction of disease-associated phenotypes are used as output metrics to screen for efficacy of gene and small molecule therapeutics. If efficacy is shown in vitro, drugs can be directed through traditional drug validation pipelines including animal studies, with the goal of delivering the novel drug to patients. This system is expected to have better translational outcomes than global drug discovery because the patient’s own cells were used to discover the phenotype modifying drug to be delivered back to the same patient.

To model a specific neuromuscular disease in 3D engineered muscle tissues (EMTs), iPSCs harbouring the disease-causing mutation are differentiated into skeletal muscle progenitors (myoblasts) by artificially mimicking the sequential stages of embryonic muscle development with a regimen of small molecules. Matched pairs of mutant and normal myoblasts are then seeded at high density in casting troughs in a 24-well format. Myoblasts are driven to secondary myogenesis to generate contractile skeletal muscle myofibers with highly organized sarcomeres (EMTs) then subjected to repeated measures on the Mantarray Platform (Curi Bio, Inc. Seattle, WA) [23]. Mantarray consists of two posts, one rigid and one flexible with a small magnet embedded at the end of the flexible post. As the EMT contracts under broad field electrical stimulation, a GMR sensor positioned outside the well detects alterations in the magnetic flux to measure post deflection. Contractile force can be precisely calculated in real time based on the stiffness of the flexible post and the amount it deflects. The major advantage of the Mantarray system is its ability to perform non-invasive, long-term longitudinal studies under a range of electrical stimulation parameters to measure the kinetics of contraction and relaxation over time (twitch, tetanus, fatigue, force-frequency relationships, calcium handling, etc). These measures provide insights into the molecular drivers of muscle pathology resulting from disease-causing mutations and an opportunity to identify new drugs with the potential to ameliorate or even reverse phenotypes in vitro.

Alec Smith presented on characterising mutant *ADSS1* deficient phenotypes in human iPSC-derived skeletal muscle. ADSS1 deficient iPSC lines have been created from *two* ADSS1 myopathy siblings, a non-diseased familial control, and isogenic variants introduced via CRISPR mutation. To build engineered muscle tissues in the Mantarray system, cells are suspended in a fibrinogen hydrogel. Myofibers are not as tightly packed as in native muscle, but forcing more fibres into the artificial matrix does not necessarily increase force production. Serum starvation is used to differentiate myoblasts into contractile myotubes. Notably, it was reported that variant cells do not differentiate as well as control lines in vitro, although further experimentation is required to confirm this preliminary observation. Force production is very low in ADSS1 variant 3D-EMTs and this is not particularly surprising given fusion capacity is poor. 3D-EMT cross sections show reduced myosin heavy chain staining. Seahorse extracellular flux was used to assess metabolism in 3D-EMTs. Basal and maximum uncoupled oxygen consumption is reduced in variant 3D-EMT’s relative to control.

David Mack suggested deriving myoblasts from the post-embryonically lethal KI mouse muscle at day 5, just prior to lethality to develop and test in the Mantarray system. Cara Timpani suggested that metabolomic analyses be performed on culture media to see which metabolites were effluxing from muscle fibres/myoblasts during Mantarray and Seahorse experiments.

Professor Wendy Hanna-Rose, Ph.D. presented on modelling ADSS1 deficiency in *C. elegans.* As a model, *C. elegans* has significant advantages through being genetically tractable, inexpensive, transparent and self-fertilising with a short lifecycle, defined developmental lineage, and capability for quantifying complex behaviour [24] as shown previously in the context of purine deficiencies for *Adsl* deficiency [25]. *C. elegans* has only one *Adss* gene, *Adss-1*, which is 50% identical to human *ADSS1*. Professor Hanna-Rose’s lab generated an *Adss-1* knock down model using RNA silencing (RNAi; double stranded *Adss1* knock down). RNAi is fed to L1 larvae *C. elegans* and a post-embryonic reduction-of-function model is recapitulated 2 days later. The team also examined the *C. elegans* KO-consortium CRISPR-engineered loss of function model null allele where the four-exon *Adss-1* gene is deleted, except for the last non-coding portion of exon 4, and replaced with a green fluorescent protein (GFP) gene. Homozygous mutants are sterile, so the strain is maintained as a heterozygote and the predicted 25% homozygosity emerges in progeny. Animals are profiled using mobility studies, LC-MS characterisation of the metabolome and imaging.

*Adss-1* mutant homozygotes are small, slow and uncoordinated. Body length and area is lower in loss- and reduction-of *adss-1* function *C. elegans* and is lower with loss- compared with reduction-of function. In swimming studies, loss or reduction of *Adss-1* reduces thrashing rate and distance travelled, and results in an abnormal curling movement. Swimming and crawling speed are reduced in both models and the effect is stronger with loss- compared to reduction-of-function. LC-MS metabolomics revealed lower succinyl AMP/adenylosuccinate but no change in IMP, AMP or GMP concentration in the reduction of function model. There was no effect of *adss-1* deficiency on glycolysis metabolites. Fumarate and malate within the mitochondrial tricarboxylic acid (TCA) cycle were both reduced. Lipid storage was assessed using BODIPY fluorescence and was more abundant in the reduction of function model than controls. Muscle structure/organisation of muscle fibres was assessed using a translational GFP fusion with the myosin heavy chain gene, allowing visualization of myosin heavy chain. Focal areas of muscle fibre breakage were evident. Neuromuscular junction function was interrogated using levamisole as a cholinergic agonist to assess muscle response to the post-synaptic potential, which was normal in reduction-of- *Adss-1* function *C. elegans*. Responsivity to aldicarb was used to assess the pre-synaptic action potential and reduction of function animals were sensitive, inferring higher levels of acetylcholine at the neuromuscular junction.

In summary, both the reduction and loss of *Adss-1* function *C. elegans* model show growth, development and fertility problems, impaired purine metabolism, mitochondrial and lipid catabolism pathways, and loss of temporal and spatial mobility. Professor Hanna-Rose acknowledged the contributions of Latisha Franklin, Rishika Patil, Melinda Jin, Belle Barriga, Maia Pappadakis and Sabrina Sony. The post presentation discussion focused on the necessity of *Adss-1* for purine homeostasis. Hyder Jinnah was interested that *Adss-1* KO in *C. elegans* is not fatal since there is only one gene. These data suggest some compensatory mechanism for its loss. David Mack questioned whether the “broken” MYO3 + fibers might indicate a hypercontractile phenotype that the extracellular matrix cannot cope with, leading to breakage of muscle composition. Wendy Hanna-Rose indicated that the breakage is mild and focal compared to *Adsl-1-* deficient *C. elegans* phenotype. Alan Beggs wondered whether the imaging indicates impaired muscle formation rather than breakage. Further characterisation is needed to confirm some of these aspects.

### Session 3: Therapeutic strategies for ADSS1 myopathy

Professor Hyder Jinnah, M.D., Ph.D. and Associate Professor Emma Rybalka, Ph.D. presented on disease biochemistry and potential therapeutics for translation. ADSS is required for generating adenylosuccinate (S-AMP) as an intermediary for maintaining the stochiometric ratio of high energy purines, AMP (0.1mM), ADP (4mM) and ATP (24mM) [26, 27]. Since muscle consumes large amounts of ATP during excitation-contraction coupling, and ADSS1 falls in the direct pathway producing these purine nucleotides (see Fig. 1), it is hypothesised that ADSS1 patients may suffer purine shortages, especially during times of high purine turnover e.g., during exercise, nutrient deprivation and growth. To date, relative concentrations of high energy purines (ATP, ADP, AMP) have not been measured in muscle from patients with ADSS1 myopathy. Assessing human muscle biopsies may be challenging due to the timelapse between biopsy and sample freezing in which there is rapid ATP degradation. Interrogating animal models might be more useful because muscles can be freeze clamped in vivo enabling a snapshot of physiological purine ratios. Phosphorus magnetic resonance spectroscopy (P-MRS) is a non-invasive method that could be employed to measure ATP and related metabolites in patient muscles [28].

According to the Human Protein Atlas (proteinatlas.org), ADSS1 is almost exclusively expressed in skeletal and cardiac muscle, while ADSS2 is expressed broadly across all tissues, but at much lower levels compared to ADSS1 in striated muscles. This distribution of these two isoforms with similar function probably explains why ADSS1 is a muscle-specific disorder. These isozymes might also be functionally distinct since ADSS1 is located cytosolically and ADSS2 on the mitochondria (reportedly), and they are primarily activated and inhibited by different substrates [29–33]. Formation of the purinosome, a dynamic protein-protein interaction network involving *de novo* purine biosynthesis enzymes as well as ADSS has been demonstrated during purine depletion as a way to rapidly expand purine (especially ATP) concentration [34, 35]. The purinosome can co-localise to mitochondria suggesting ADSS2 or the heterodimerisation of ADSS1 with ADSS2 might be necessary for purinosome assembly and function. Purinosome assembly has not been studied in ADSS1 myopathy. If ADSS2 is the predominant isozyme involved in its assembly and function, overexpression of ADSS2 could be therapeutic (Fig. 1, target 1), although there is a critical need for understanding how purine biology is affected by ADSS1 deficiency to inform directed therapeutic targeting.

Loss of ADSS1 function limits muscular capacity for purine metabolism (e.g., IMP, hypoxanthine, xanthine, adenosine, inosine) causing their excretion from muscle via xanthine oxidoreductase (XOR) and reduced AMP biosynthesis (Fig. 1, Target 2). Allopurinol and febuxostat are clinically utilised XOR inhibitors [36] that could be useful for ADSS1 myopathy to stem this excretion. Blocking AMPD from metabolising AMP to IMP (Fig. 1, Target 3), or adenosine deaminase (ADA) from metabolising adenosine to inosine, may also be useful to stem the flux of purines from muscle. Alternatively, exploiting recycling pathways of adenosine (i.e., S-adenosylmethionine, or SAM; Fig. 1, Target 4) and/or adenine (Fig. 1, Target 4) could be therapeutic. Stemming adenine degradation into 2,8-dehydroxyadenine through dual treatment of adenine/adenosine with a XOR inhibitor (Fig. 1, Target 5), could be especially so. An underappreciated function of ADSS1 is functional coupling between cytosolic purine metabolism and mitochondria, and loss of ADSS1 function would disrupt this coupling by reducing aspartate sequestration and fumarate synthesis (Fig. 1). Re-purposed fumarate esters (Fig. 1, Target 6) could be useful to manage this aspect [37, 38] as well as inducing broad spectrum cytoprotection against metabolic, inflammatory and oxidative stress [39, 40]. Supplemental ATP (Fig. 1, Target 7) was used by one patient from the Indian ADSS1 myopathy patient cohort. However, extracellular ATP is likely to be rapidly dephosphorylated and is probably mostly consumed by the liver during first pass metabolism so is unlikely to augment muscle ATP levels.

Understanding the cause of weakness in ADSS1 myopathy is necessary. It is easy to presume that lack of ATP is the primary cause of weakness. However, muscle deconditioning, pathology (e.g., vacuole and nemaline inclusions, fatty infiltrate) and wasting/loss likely contribute. Weakness due to deconditioning is probably fixable in the short-term, muscle pathology may be fixable over the moderate term, but muscle loss is probably permanent. There are key questions that need to be answered to adequately assess potential therapeutics for ADSS1 myopathy: (1) what duration of therapy is required to assess efficacy? and (2) what biomarker(s) could measure “target engagement” to ensure the correct treatment path has been taken? In this context, the goal of therapy must be considered. A very effective therapeutic may not be able to reclaim lost strength, but it may be able to prevent further strength decline. This is an important consideration for future clinical trials that measure treatment efficacy.

The post-talk discussion focused on the use of P-MRS to assess purine homeostasis in patients. Published reports indicate this method can measure ATP and PCr in muscles from living human individuals. However, this method is not widely available. Matt Hall indicated that hyperpolarized carbon 13 ([13]C) MRI technology may also be useful [41]. Jeff Brault agreed that previous [13]C enrichment nuclear magnetic resonance (NMR) methods required large voxels resulting in low resolution, but this has changed now with hyperpolarization methods. These types of methodologies could be useful in clinical trials to confirm experimental drugs are engaging their targets. Emma Rybalka asked whether there was a recommended trial duration for measuring outcome measures in purine metabolism disorders of muscle. Hyder Jinnah thought that duration may depend on a patient’s functional state when starting a trial. Patients with mild exercise intolerance may show benefits more quickly, while those with significant myofiber damage or loss may show benefits more slowly, if at all. Patients need to be carefully matched to the outcome variable that is intended for use. Emma Rybalka questioned how an informed outcome measure could even be selected for an ultra-rare NMD trial given trials are likely to be *n* of 1 or 2. There would need to be a good understanding of the patients’ unique symptomology and progression rate. David Mack highlighted that natural history studies are so important in this regard, yet they take decades and huge funding. Alan Beggs indicated that many of the NMD function-based assessments used in clinical trials are not undertaken clinically as part of retrospective natural history studies, indicating a disconnect between available natural history data and clinical trial design. There was group discussion regarding serum CPK as a biomarker of muscle degeneration. Activity of this enzyme is not uniformly increased across ADSS1 myopathy patients. Even in Duchenne patients for whom CPK activity is a disease biomarker, loss of muscle mass in late disease stages often leads to rapid decreases in enzyme units even though muscle degeneration persists.

There was also focused discussion on whether exercise might exacerbate ADSS1 myopathy. Application of even a mild exercise stressor might cause significant purine excretion from muscle. However, reducing physical activity to such an extent that disuse muscle wasting ensues could also be problematic. Ultimately, the rate of progression of the myopathy might depend on the balance of the various degraded purines retained and emitted from muscle. Adenosine and inosine have purported pro- and anti-inflammatory properties, respectively, and XOR generates reactive oxygen species during metabolism of inosine and xanthine based on the conformation of the enzyme complex, which changes in response to local inflammatory signals. Understanding in which contexts these metabolites are affected in ADSS1 myopathy, and whether alterations are a primary driver of myopathy, requires intensive study.

Dr Behzad Moghadaszadeh, Ph.D. and Professor Alan Beggs, Ph.D. presented on the development of AAV-based gene replacement therapies for ADSS1 myopathy. The advantages of AAV-mediated gene transfer are (1) low immunogenicity and good biosafety; (2) reasonably good bioavailability and delivery to striated muscles; and (3) it can mediate long-term gene delivery in vivo. Drawbacks include: (1) emerging evidence for occasional immune-mediated toxicity associated with high dose AAV delivery, (2) limited cargo capacity – which is not a problem for the small *ADSS1* gene; and (3) possibility of generating neutralizing antibodies against the AAV or cargo. Plasmids encoding viral genes, and the recombinant gene of interest are co-transfected to produce recombinant AAV that is replication deficient but carries the gene of interest.

For initial proof of concept concert studies, a traditional AAV9 serotype vector with good muscle tropism was designed utilizing the synthetic MHCK7 promoter to drive gene expression of the full length human *ADSS1* transcript in murine skeletal and cardiac muscle. Native human *ADDS1* and codon-optimised human *ADSS1*, which is expected to induce faster and more efficient protein expression [44], were generated. *Adss1* KO mice were injected retro-orbitally at 4 weeks age and sacrificed 2 weeks post injection. In a preliminary study, both native and optimised AAV transgenes induced ADSS1 protein expression in skeletal (quadriceps and diaphragm) and cardiac muscles. Ongoing experiments include measuring ADSS1 enzyme activity to assess functional restoration, and optimising muscle targeting using muscle-directed AAV capsids (e.g., myoAAVs) [44]. Compared to AAV9, myoAAV-mediated transgene delivery has previously been shown to provide superior therapeutic benefits at lower doses than required for AAV9 legacy vectors. Future experiments will use AAV-*ADSS1* to demonstrate whether *Adss1*-KI lethality is due to *Adss1* D261N (if AAV-ADSS1 rescues the KI phenotype) or is unrelated or due to a dominant negative effect (no rescue of the compound heterozygous KO x KI phenotype).

Post-presentation discussion focused on delivery versus the turnover of muscle tissue resulting in longevity of muscle targeted gene therapy. David Mack pointed out that in the canine model of X-linked myotubular myopathy, therapeutic benefits of gene therapy lasted > 10 years.

Dr Matt Hall presented on considerations for creating a drug discovery strategy for treating ADSS1 myopathy, and the NCATS high throughput small molecule drug screening platform for rare diseases. Hundreds of thousands of molecules can be screened with minimal biological material. There is an NCATS Pharmaceutical Collection (NPC) that can be screened against any disease biology (where there is a difference between a biological phenotype and WT) for rapid translational drug repurposing [45]. Complexity of a given model limits the speed of the screening but not ability. Drug discovery assays could be created using multicellular models such as *C. elegans*, single cell or enzymatic approaches. Multiple clinical trials have eventuated using this approach.

**Fig. 2:**
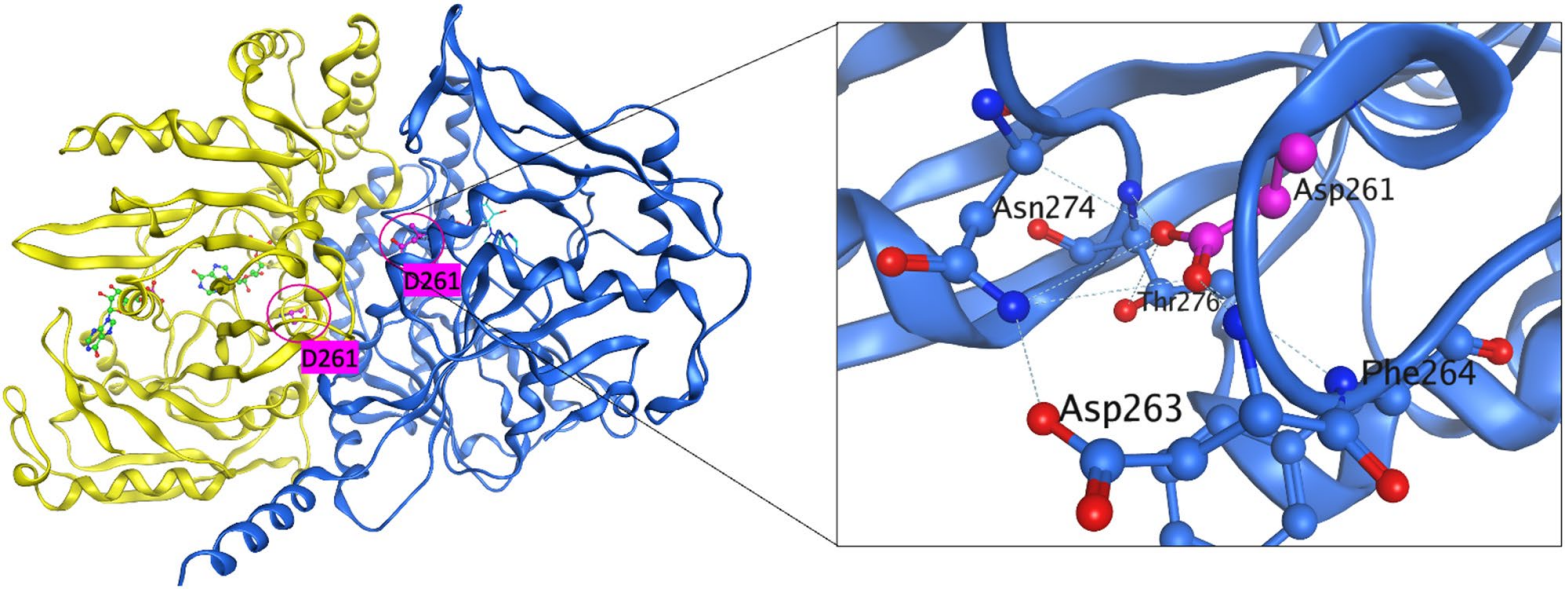
Crystallographic structure of ADSS1. Left panel: Ribbon representation of the mouse ADSS1 co-crystal structure illustrating the dimer formation (PDB code: 1IWE). The monomers are shown in yellow and blue; the substrate/cofactor is shown in green using ball and stick mode. D261 is located at dimer interface which is > 9Å away from the active site. Right panel: Detailed view of the D261 with surrounding residues. Potential hydrogen-bonds are labeled with dotted lines. All molecular depictions were prepared using MOE molecular modeling software (http://www.chemcomp.com/)

Therapeutic development for ADSS1 myopathy could take many approaches. The complexity of the biology that stems from a single ADSS1 point mutation is not yet defined. One hypothesis is that the biochemical activity of the enzyme is modulated in a way that could be restored from a biochemical or protein point of view, either by directly screening for biochemical activators, or screening for a cellular phenotype approach that modifies the ADSS1 deficient cellular metabolomics. There is no crystal structure of human ADSS1, but there is a mouse structure that exists and the identity between the two is quite strong. Preliminary modelling reveals that the Asp261Asn is at the interface of dimerization of the two monomers (Fig. 2). It is possible that there are implications for heterodimerisation between ADSS1 and ADSS2, and that dimerization might be requisite for biochemical activity and facilitated through post-translational modification. Purified WT and mutant ADSS1 has been generated at NIH and is freely available to researchers on request. NCATs scientists have also generated a high throughput-amenable mass spectrometry assay for measuring the reaction substrates and products of ADSS: IMP, aspartate, S-AMP/ASA and AMP for cell or biochemical based screening.

Matt Hall acknowledged the NCATS DMPK team led by Dr. Xin Xu, who are supporting pre-clinical studies. Post presentation discussions centred around use of the targeted mass spectrometry [13]C metabolite assay to screen patient-derived iPSC myoblasts from David Mack’s lab. Fluorescent ATP and GTP sensors could be used to assess endpoint purine recovery. Wendy Hanna-Rose asked whether high-throughput drug screening platforms are likely to pick up very different hits based on whether an enzymatic or more complex functional model (e.g., *C. elegans*) is used. Matt Hall indicated that he was open to trying several approaches, but a general challenge with screening is selecting a proximal approach, for example biochemical assays versus phenotypes. In most cases, understanding of the disease, and the therapeutic hypothesis, will prioritise a single approach.

### Session 4: Biomarkers identification

Dr Jaymin Upadhyay, Ph.D. presented on identifying non-invasive musculoskeletal biomarkers in ADSS1 myopathy. Dr Upadhyay’s team used Electrical Impedance Myography (EIM) in a pilot study in ADSS1 myopathy [13]. EIM is a non-invasive technology that administers a painless electrical signal from the skin surface to underlying muscle tissue to evaluate muscle integrity. EIM has multiple advantages over, for example, MRI, in that the method can be utilized in a variety of settings (i.e., at-home), used to assess multiple muscle groups throughout the body, and is a cost-effective and portable technique. EIM has capability to detect diseased muscle tissue, track disease progression and monitor treatment effect in multiple neuromuscular and musculoskeletal conditions [46–50]. EIM has also been used as a biomarker to monitor progression of fibrodysplasia ossificans progressiva (FOP), a disease where soft tissues such as muscle and tendons develop into heavy bone. Traditionally, PET, CT and MRI have been used to monitor FOP progression which are associated with high cost and exposes patients to radiation. The study began just as the COVID pandemic onset, which required the team to think outside the box in terms of data acquisition, as in-person study visits were not a possibility. An at-home EIM acquisition paradigm was developed by the study team, where the EIM devices were sent to individuals with FOP and hosted virtual training and data acquisition sessions to be conducted in the patients’ home. The study showed robust differences in EIM measures with increased disease progression [50]. EIM is also currently being used within a deep phenotyping study of infantile and late-onset Pompe disease (IOPD/LOPD) patients. In addition to EIM, muscle (quadriceps) MRI, blood proteomic biomarkers, patient reported outcome measures, and motor assessments (walk (4 m, 2 min), balance, grip, 9-hole pegboard, speech production) are assessed. Preliminary EIM data collected in patients with IOPD/LOPD suggest this methodology may represent an objective and non-invasive biomarker for monitoring muscle healthy in this lysosomal storage disease.

To date, three ADSS1 myopathy patients have undergone evaluation with EIM (15-year-old ambulatory male, 20-year-old ambulatory female, 63-year-old non-ambulatory male) [13]. All participants carry a homozygous pathogenic variant in *ADSS1* c.910G > A (p.Asp304Asn). Relative to heterozygous controls, patients with ADSS1 myopathy demonstrated lower phase and reactance values at 100 kHz, while muscle resistance was higher, which together points to the presence of diseased muscle tissue in the ADSS1 cohort. There is good consistency between the three ADSS1 myopathy patients for EIM readouts. Future directions for ADSS1 myopathy will include patient and caregiver experiences via focus group evaluations and individual interviews as was recently conducted by Dr Upadhyay’s group for Niemann Pick Disease Type C [51]. Deep phenotyping evaluations of patients with ADSS1 will also be undertaken, which includes clinical evaluation, behavioural testing (motor, speech production, cognition), musculoskeletal MRI, EIM, neuroimaging and blood proteomics to identify good biomarkers of disease progression.

Post-presentation discussion centred on increasing patient availability to the deep phenotyping study and matching the patient deep phenotyping study readouts to pre-clinical mouse studies, including EIM. Alan Beggs wondered if the EIM readouts could pick up pathologies that might not be visible through histopathology analysis of mouse tissues. It is possible that fat especially, is washed out of histological sections during staining e.g., with ethanol, which could be detected in vivo through EIM studies. Hyder Jinnah questioned the reproducibility of EIM across different muscles even in healthy patients. Recent work has indeed shown that EIM is a reproducible measure of muscle health [52].

Dr Jeff Brault, Ph.D. presented on adenine nucleotide measurement in NMDs. ATP is needed as an energy source in muscle to drive synthesis of genetic materials, macro molecules, and cellular components; muscle movement; molecule transportation and electrical energy. The energy tproduced by ATP is not dependent upon the amount of ATP in the cell, but rather the ratio of metabolic products to reactants of ATP hydrolysis e.g., the ATP: ADP ratio [53]. However, there are other uses for ATP including (1) intracellular signalling as a substrate for kinases; (2) cell-to-cell communication in purinergic signalling; (3) DNA and RNA; and (4) protein solubility in which ATP reduces aggregation of proteins (for review of point 4 see [54]). These processes are dependent only on the concentration of ATP. The ATP/ADP ratio and ATP concentration are controlled independently. The adenine nucleotide pool is well defended in skeletal muscle, even during intense exercise. ATPases, oxidative phosphorylation, glycolysis, CPK and myoadenylate kinase control the ATP: ADP ratio. However, efficiency of enzymes of purine metabolism, as well as the rate at which cell processes draw on purines (e.g., DNA and RNA biosynthesis, protein binding (i.e., to filamentous actin) and guanine nucleotide biosynthesis) control the ATP concentration. Simultaneously assessing ATP, alongside lower order purine catabolites such as ADP, IMP, UA, hypoxanthine, xanthine, AMP, adenine, inosine, and adenosine is important to provide a complete metabolic picture.

The Brault lab has developed an ultra-performance liquid chromatography method to quantitate purines and their degraded products to < pmol/sample in ~ 10 min, in which identity/purity is confirmed via retention time, absorbance spectra and mass [55]. Guanines and deoxy forms of nucleotides can also be assessed [56]. To date, the methodology has been used to assess mitochondrial myopathy in succinyl-coA deficient mouse muscle. Ex vivo muscle contraction studies revealed loss of function of slow-twitch (oxidative) soleus muscle, but fast-twitch (glycolytic) EDL muscles are unaffected. ATP levels are unaffected between controls and mutants. However, AMP levels are increased in both EDL and soleus and ADP levels are increased in EDL, indicating shifts in energy availability based on purine ratios (Lancaster et al.., manuscript submitted).

Muscles derived from 10.5-month-old *Adss1* KO (*n* = 2) and normal control (*n* = 2) mice from the Beggs lab were assayed. Non-contracted TA (mixed), EDL (predominantly glycolytic) and soleus (predominantly oxidative), and contracted EDL and soleus, were flash-frozen and assessed. ATP and ADP levels were comparable in *Adss1* KO TA compared to WT controls. However, AMP was reduced, and IMP was increased. GTP was also increased in *Adss1* KO TA. There was no difference in the ATP/ADP ratio or the sum of nucleotides. In non-contracted EDL, ATP, ADP, AMP and GTP levels were comparable between *Adss1* KO’s and normal controls. However, IMP levels were ~ 3-fold higher. Again, there were no differences in the ATP: ADP ratio or the sum of nucleotides. Comparatively in the contracted EDL, ATP and GTP levels reduced, but ADP, AMP and especially IMP levels were increased, resulting in reduced ATP: ADP ratio but no change to nucleotide sum. This effect seems relatively comparable between *Adss1* KO and normal EDL. In uncontracted soleus, ATP levels are reduced, while IMP and GTP levels are increased in *Adss1* KO’s compared to normal. Both the ATP: ADP ratio and the nucleotide sum appear reduced in *Adss1* KO muscles compared to normal. Comparatively, in contracted soleus, ATP was reduced and GTP increased in *Adss1* KO’s relative to normal, however ADP, AMP and IMP were comparable. Again, both the ATP: ADP ratio and the nucleotide sum were reduced in *Adss1* KO soleus. In summary, ATP: ADP ratio and ATP concentration are controlled independently and in *Adss1* KO muscle, IMP concentration may be greater in all muscles supporting slowed activity of ADSS1. Alternatively, ATP concentration and ATP: ADP ratio may be reduced in soleus but not EDL and TA, suggesting fibre type and/or metabolic (oxidative versus glycolytic) differences. GTP concentration may also be greater in soleus and TA supporting a shift in adenine/guanine ratio. Future work will incorporate more samples and interrogate these metabolic shifts further.

Post-presentation discussion centred around fibre-type differences between species (mouse and human) and muscles (i.e., in mice, EDL versus soleus). Slow-twitch oxidative muscle could be predominantly impacted if reactive oxygen species are driving pathology in ADSS1 myopathy. Clearly, measuring singular nucleotides is insufficient to assess disease pathogenesis. Metabolic fluxes must be considered through the purine/guanine metabolism and degradation pathways. Jeff Brault indicated that simply euthanizing an animal and collecting tissues will result in large fluxes of purines through the degradation pathways and artifactually low ATP levels. Emma Rybalka suggested that degradation metabolites (e.g., inosine, hypoxanthine, xanthine) need to be analysed both in muscles and plasma/cell media. Hyder Jinnah indicated that tissue treatment with perchloric acid inactivates metabolic enzymes and preserves purines.

## Summary and strategic next steps

The meeting addressed the current state of knowledge and research needs concerning ADSS1 myopathy pathogenesis, clinical presentation, biomarker discovery and therapeutic development. An ADSS1 Myopathy Research Consortium was established from the meeting and key action points and deliverables of the group include:

- Creation of consortium terms of reference, guidelines and tools to streamline sharing of research data and resources. Regular quarterly meetings will be actioned to maintain consortium synergy and update on developments.
- Standardisation of clinical assessments across the geographically diverse patient cohorts for synergy and patient registry integrity. A specialised workshop for clinicians is being organised to facilitate this aspect.
- Understanding the disease pathophysiology and thorough phenotyping of animal and cell models that recapitulate human disease phenotypes is crucial for identifying translational therapeutics and disease biomarkers. Members of the consortium are conducting natural history studies to address this knowledge gap.
- Establishing standard operating procedures that use clinically-compatible pre-clinical assessments are critical to ensure robust translational outcomes for therapeutic development of gene and small molecule strategies. The consortium will create these documents, and they will be made available on the Cure ADSSL1 website for global accessibility.
- Preclinical evaluation of metabolic replenishment therapies. Members of the consortium are seeking funding to conduct in vitro and in vivo screens of a variety of therapeutics.

## Conference participants

### In-person presenters (in presenter order)

- Naveen Baweja, Cure ADSSL1, Los Angeles, USA.
- Priyanka Kakkar, Cure ADSSL1, Los Angeles, USA.
- Matthew Hall, Ph.D., Scientific Director, Division of Preclinical Innovation, National Institute of Health, NCATS, Rockville, USA.
- Alan Beggs, Ph.D., Director, Manton Centre for Orphan Disease Research, Boston Children’s Hospital, Harvard Medical School, Boston, USA.
- Behzad Moghadaszadeh, Ph.D., Senior Research Fellow, Manton Centre for Orphan Disease Research, Boston Children’s Hospital, Harvard Medical School, Boston, USA.
- David L. Mack, Ph.D., Associate Professor, Institute for Stem Cell and Regenerative Medicine, University of Washington, Seattle, USA.
- Wendy Hanna-Rose, Ph.D., Professor of Biochemistry and Molecular Biology, The Pennsylvania State University, University Park, USA.
- Hyder Jinnah, M.D., Ph.D., Professor, Department of Neurology, Human Genetics and Pediatrics, Emory University, Atlanta, USA.
- Emma Rybalka, Ph.D., Associate Professor, Institute for Health and Sport and Australian Institute for Musculoskeletal Science, Inherited and Acquired Myopathies Program, Victoria University, Melbourne, AUS.
- Jaymin Upadhyay, Ph.D., Assistant Professor, Department of Anaesthesiology, Critical Care and Pain Medicine, Boston Children’s Hospital, Boston, USA.
- Jeff Brault, Ph.D., Associate Professor of Anatomy, Cell Biology & Physiology, Indiana University School of Medicine, Indianapolis, USA.

### Remote presenters (in presenter order)

- Hyunh Jun Park, M.D., Ph.D., Clinical Associate Professor, College of Medicine, Yonshei University, Seoul, South Korea.
- Nalini Atchayaram, M.D., Ph.D., Professor, Department of Neurology, National Institute of Mental Health And NeuroSciences (NIMHANS), Bengaluru, India.
- Dipti Baskar, M.D., Department of Neurology, National Institute of Mental Health And NeuroSciences (NIMHANS), Bengaluru, India.
- Hacer Durmas, M.D., Ph.D., Department of Neurology, Istanbul Faculty of Medicine, Istanbul University, Istanbul, Turkey.
- Perry Shieh, M.D., Ph.D., Professor of Neurology and Pediatrics, University of California Los Angeles, Los Angeles, USA.
- Yang Xia, M.D., Ph.D., Professor, National Medical Metabolomics International Collaborative Research Center, Xiangya Hospital, Central South University, Changsha, China.
- Linlin, Wan, M.D., Department of Radiology, Xiangya Hospital of Central South University, Changsha, China.
- Alec Smith, Ph.D., Assistant Professor, Institute for Stem Cell and Regenerative Medicine, University of Washington, Seattle, USA.

### Participants (non-presenting in attendance, in alphabetical order)

- Belle Barriga, The Pennsylvania State University.
- Ken Cheng, Ph.D., Division of Preclinical Innovation, NCATS, Rockville, USA.
- Kevin Green, Dystrophy Concepts Pty. Ltd, Indianapolis, USA.
- Melinda Jin, The Pennsylvania State University.
- Min Shen, Division of Preclinical Innovation, NCATS, Rockville, USA.
- Pranav Shah, Ph.D., National Institutes of Health, NCATS.
- Tom Bonsett, Dystrophy Concepts Pty. Ltd, Indianapolis, USA,
- Xin Xu, Ph.D., National Institute of Health NCATS.

### Participants (non-presenting remote, in alphabetical order)

- Amy Wang, Ph.D., National Institute of Health, NCATS, USA.
- Angela Lek, Ph.D., Vice President for Research, Muscular Dystrophy Association, USA.
- Angelo D’Alessandro, Ph.D., University of Colorado, USA.
- Bo Qi, Institute for Health and Sport, Victoria University, Melbourne, AUS.
- Cara Timpani, Institute for Health and Sport, Victoria University, Melbourne, AUS.
- Casie Genetti, M.S., C.G.C., Harvard University, Boston Children’s Hospital, USA.
- Didier Debrincat, Institute for Health and Sport, Victoria University, Melbourne, AUS.
- Dongsheng Guo, University of Massachusetts, USA.
- Emily Hickey, Harvard University, Boston Children’s Hospital, USA.
- Evrim Atas, Ph.D., Director, Research Portfolio, Muscular Dystrophy Association, USA.
- Fabrizio Pertusati, Ph.D., School of Pharmacy and Pharmaceutical Sciences, Cardiff University, UK.
- Guinevere Spiesberger, Institute for Health and Sport, Victoria University, AUS.
- Heike Kölbel, M.D., Ph.D., Department of Neuropediatrics, University Hospital Essen, DEU.
- Merve Koç Yekedüz, M.D., Ph.D., Harvard University, Boston Children’s Hospital, USA.
- Monkol Lek, Yale University School of Medicine, USA.
- Nate Hoxie, National Institutes of Health, NCATS, USA.
- Ovid Nativi, National Institutes of Health, NCATS, USA.
- Raquel van Gool, Ph.D., Harvard University, Boston Children’s Hospital.
- Rich Horgan, Chief Executive Officer, Cure Rare Disease, USA.
- Rishika Patil, The Pennsylvania State University, University Park, USA.
- Rodney Kellems, Ph.D., The University of Texas, Houston, USA.
- Ryan Bagaric, Institute for Health and Sport, Victoria University, Melbourne, AUS.
- Saraswati Nashi, M.D., NIMHANS, Bangalore, IND.
- Shushu Huang, M.D., Ph.D., Yale University School of Medicine, USA.
- Stephanie Kourakis, Institute for Health and Sport, Victoria University, Melbourne, AUS.
- Thomas Yates, Institute for Health and Sport, Victoria University, Melbourne, AUS.

## Data Availability

The preliminary datasets presented in this published article are not publicly available but are available from the corresponding authors on reasonable request.

## Abbreviations

3D: Three dimensional
^13^C: Carbon thirteen
AAV: Adeno-associated virus
ADA: Adenosine deaminase
ADP: Adenosine diphosphate
ADSS: Adenylosuccinate synthetase
ADSS1/ADSSL1: Adenylosuccinate synthetase isozyme (like) 1 (striated muscle)
ADSS2: Adenylosuccinate synthetase isozyme 2
ADSL: Adenylosuccinate lyase
AMP: Adenosine monophosphate
AMPD1: Adenosine monophosphate deaminase 1
ATP: Adenosine triphosphate
BIPAP: Bilevel positive airways pressure
Cas9: CRISPR associated protein 9
COX: Cytochrome c oxidase
CPAP: Continuous positive airways pressure
CPK: Creatine phosphokinase
CRISPR: Clustered regularly interspaced short palindromic repeats
EDL: Extensor digitorum longus
EIM: Electrical impedance myography
EMG: Electromyography
EMT: Engineered muscle tissue
FOP: Fibrodysplasia ossificans progressiva
GMP: Guanosine monophosphate
GTP: Guanosine triphosphate
GMR: Giant magnetoresistance
IMP: Inosine monophosphate
iPSC: Inducible pluripotent stem cell
KO: Knock-out
KI: Knock-in
LC-MS: Liquid chromatography-mass spectrometry
MRI: Magnetic resonance imaging
MYO3+: Myosin heavy chain isoform 3 positive
Myo-AAV: Muscle directed-adenoviral associated vector
NADH: Nicotinamide adenine dinucleotide (reduced form)
NCATS: National Center for Advancing Translational Science
NIH: National Institutes of Health
NMD: Neuromuscular disease/disorder
NMR: Nuclear magnetic resonance
NPC: National Center for Advancing Translational Science Pharmaceutical Collection
P-MRS: Phosphorous magnetic resonance spectroscopy
PNC: Purine nucleotide cycle
RNA: Ribonucleic acid
RT-PCR: Reverse transcription-polymerase chain reaction
SAM: S-adenylmethionine
TCA: Tricarboxylic acid
USC: Urine-derived stem cells
WT: Wild type
XOR: Xanthine oxidoreductase
